# Hybrid Selection Method of Feature Variables and Prediction Modeling for Municipal Solid Waste Incinerator Temperature

**DOI:** 10.3390/s21237878

**Published:** 2021-11-26

**Authors:** Jingcheng Guo, Aijun Yan

**Affiliations:** 1 Faculty of Information Technology, Beijing University of Technology, Beijing 100124, China; yanaijun@bjut.edu.cn; 2 Engineering Research Center of Digital Community, Ministry of Education, Beijing 100124, China; 3 Beijing Laboratory for Urban Mass Transit, Beijing 100124, China

**Keywords:** municipal solid waste, incinerator temperature prediction, feature selection, stochastic configuration networks

## Abstract

It is difficult to establish an accurate mechanism model for prediction incinerator temperatures due to the comprehensive complexity of the municipal solid waste (MSW) incineration process. In this paper, feature variables of incineration temperature are selected by combining with mutual information (MI), genetic algorithms (GAs) and stochastic configuration networks (SCNs), and the SCN-based incinerator temperature model is obtained simultaneously. Firstly, filter feature selection is realized by calculating the MI value between each feature variable and the incinerator temperature from historical data. Secondly, the fitness function of GAs is defined by the root mean square error of the incinerator temperature obtained by training SCNs, and features obtained by MI methods are searched iteratively to complete the wrapper feature selection, where the SCN-based incinerator temperature prediction model is obtained. Finally, the proposed model is verified by MSW incinerator temperature historical data. The results show that the SCN-based prediction model using the hybrid selection method can better predict the change trend of incinerator temperature, which proves that the SCNs has great development potential in the field of prediction modeling.

## 1. Introduction

The main goal of municipal solid waste (MSW) incineration is to realize the resource, reduction and harmlessness of MSW [1]. During the MSW incineration process, if the incinerator temperature (generally the temperature of the primary combustion chamber) is less than 850 °C, the dioxin with strong carcinogenicity cannot be effectively decomposed, which will endanger human health [2]. Therefore, it is whether the incinerator temperature is controlled well or not that becomes a key indicator to measure whether operation of MSW incineration is normal. In order to stably control the incinerator temperature, the operation index of relevant equipment needs to be adjusted in real time according to the change trend of the incinerator temperature. However, due to the volatility of waste components and the strong lag in the incineration process [3], it is difficult for on-site operators to judge the change trend of furnace temperatures in time, which may cause the furnace temperature to be out of control. Therefore, it is of significance to establish a prediction model of furnace temperature that can accurately predict the change of furnace temperature in the incineration process.

At present, methods of modeling for the MSW incineration process are widely focused on the mechanism analysis modeling methods [4,5], and the computational fluid dynamics (CFD) technology is used to simulate and model the MSW incineration process [6,7]. Although the mechanism analysis has the advantages of high reliability and good extrapolation, the accuracy of the mechanism model is difficult to satisfy due to the complex characteristics of the MSW incineration process, such as the strong nonlinearity, the large fluctuations in waste composition, and the severe coupling between variables. However, a large amount of process industry data can be obtained with the development of sensor technology, which provides a powerful guarantee for data-driven method modeling [8]. Nowadays, data-driven modeling methods mainly include back propagation (BP) neural network, support vector machine (SVM) and stochastic configuration networks (SCNs). Nevertheless, due to well-known reasons, such as the BP neural network easily falling into local optimum and the convergence speed being slow, SVM has low training efficiency for large-scale data samples which makes the application of these typical methods have certain limitations. Due to the universal approximation property, random assignation of hidden nodes parameter and fast training speed, SCNs as an emerging data-driven modeling method [9] have attracted the attention of researchers. It has been widely applied to the field of pattern classification [10], function approximation [11] and parameter prediction [12,13].

In this paper, SCNs are employed to establish the MSW incinerator temperature prediction model, which is completed in two steps. Firstly, some unrelated variables are removed from several feature variables by the mutual information (MI) method. Then, SCNs and genetic algorithms (GAs) are combined to form a GA-SCN feature selection method where redundant variables are further eliminated. When the error of the SCN-based temperature model of the MSW incinerator reaches the specified value, not only is the hybrid selection of feature variables completed, but the SCN-based incinerator temperature prediction model is also obtained. Finally, effects of feature selection and the performance of the incinerator temperature prediction model are evaluated by historical data on incinerator temperature.

The rest of this paper is organized as follows: Section 2 introduces the MSW incineration process. Section 3 reviews the feature selection method and SCNs. Section 4 describes the establishment process of the incinerator temperature model. Section 5 conducts the experimental evaluation. In the final section, the conclusions and future research are presented.

## 2. MSW Incineration Process

**Figure 1:**
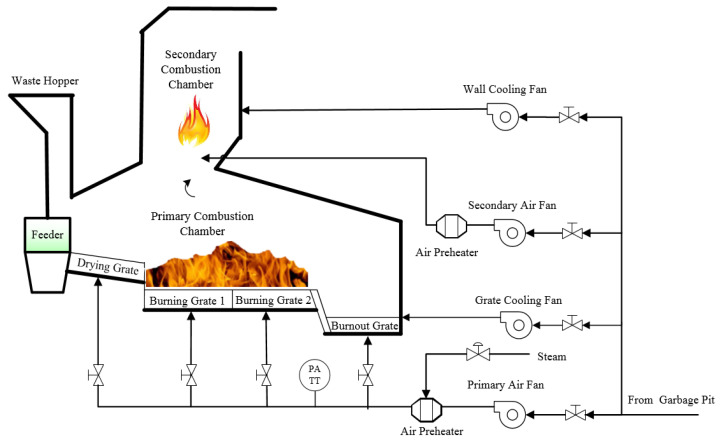
Flow chart of MSW incineration.

Taking the incinerator used by a waste incineration company in Beijing as an example, the MSW incineration process is shown in Figure 1. The process can be divided into four main sub-processes: grate speed, grate temperature, air flow and combustion chamber. The specific process is as follows:

First, the MSW is sent to the incinerator through the feeder; under the effect of high temperature radiation from the chamber and convection heat transfer of primary air, the water drying process is carried out along the direction of the drying grate. Then the dried waste, placed on the burning grate (a total of two stages, burning grate 1 and burning grate 2), is used for the precipitation of volatiles. Finally, the fixed carbon in the waste is burned to form carbon oxides in the burnout grate. At the same time, the separated volatiles are burned in a combustion chamber with oxygen from primary air, and incomplete combustion volatiles are further burned under the influence of the secondary air.

In order to avoid the MSW incineration process that produces dioxin gas with strong carcinogenicity, it is necessary to control the incinerator temperature above 850 °C, which can promote the decomposition of dioxins [14]. Therefore, the incinerator temperature should be controlled stably and precisely, which has become a key index for judging whether operation of the MSW incineration process is normal.

For the incineration process shown in Figure 1, incinerator temperature is affected by the operating conditions of grate speed, grate temperature and air flow sub-process. The feature variables that may affect the incinerator temperature are *n* = 65 in total. The grate speed sub-process includes grate speed of the drying grate, the burning grate 1, the burning grate 2 and the burnout grate, which has *n*_1_ = 18 feature variables; grate temperature sub-process includes grate temperature of the drying grate, the burning grate 1, the burning grate 2 and the burnout grate, which has *n*_2_ = 24 feature variables; the air flow sub-process includes various variables of fan, air heater, the air flow under the drying grate, the burning grate 1, the burning grate 2 and the burnout grate (mainly air volume, pressure and temperature), which is *n*_3_ = 23. The detailed variable information is shown in Table 1. Unfortunately, the large number of process variables probably include irrelevant or redundant feature variables. If the model is directly built by all variables, this may lead to high complexity, large fitting error and even over-fitting. Therefore, before the incinerator temperature prediction model is established by the data-driven method, it is necessary to select features based on the sample data.

## 3. Related Work

In this section, some related work of feature selection methods and the SCNs modelling method proposed will be introduced.

### 3.1. Feature Selection

The feature selection method can be divided into filter methods [15] and wrapper methods [16] depending on whether the classifier or the predictor directly participates in feature selection. Filter methods rank the features of the sample data by some ranking criteria, and then set the threshold to eliminate features that cannot satisfy the condition [17], such as the correlation criterion [18] and mutual information (MI) [19]. Correlation criteria calculates simply and can effectively eliminate less relevant variables. However, it can only detect linear dependencies between variable and target. Unfortunately, there are a large number of nonlinear relationships in actual sample data, so it has certain limitations. The MI-based feature selection method is based on information entropy to quantify the dependency between two variables, and features are selected by MI-based ranking. The MI definition is as shown in (1):

(1) $$I(X;Y)=\sum_{x\in X}\sum_{y\in Y}p(x,y)\log\frac{p(x,y)}{p_{X}(x)p_{Y}(y)}$$

where *x* and *y* represent the attribute values of the feature variables *X* and *Y*, respectively, *p_X_*(*x*) and *p_Y_*(*y*) represent the edge probability distribution, *p*(*x*, *y*) represents the joint probability distribution. If *X* and *Y* are independent, then *I*(*X*; *Y*) = 0; the greater *I*(*X*; *Y*) represents the higher the dependence between *X* and *Y*. MI values are obtained by (1) between feature variable and target variable. Then, required feature variables are selected by setting a MI threshold; in other words, irrelevant variables are eliminated. However, filter methods are independent of the model training process, and redundant variables may exist in the selected feature subset [20]. As a result, it may lead to the poor modeling accuracy.

Wrapper methods use the classifier or the predictor as a black box, and use the classifier or the predictor performance as the basis for evaluating whether the feature selection is effective, and the classifier or predictor can be obtained while feature selection is completed. Typical wrapper methods include sequential feature selection (SFS) algorithms [21], GAs [22] and particle swarm optimization (PSO) algorithms [23]. Although wrapper methods can effectively eliminate irrelevant variables and redundant variables, there are some limitations such as high complexity and low efficiency. The combination of filtering and wrapper methods can properly compensate for their deficiencies and improve the effect of feature selection [24,25,26], however these limitations still exist. Thus, it is necessary to select the modeling method.

### 3.2. Stochastic Configuration Networks

The stochastic configuration network, proposed by Wang et al. is a randomized method with universal approximation property [9]. Compared with traditional artificial neural networks, SCNs randomly assign the parameters of the hidden layer nodes in the light of a supervisory mechanism and evaluate the connection weight between the hidden layer nodes and the output layer nodes, which greatly avoids the influence of neural network learning ability from artificial experience, and its training efficiency also has obvious advantages. There are three learning algorithms provided by [9]. The proposed SC-III algorithm is used in this paper, whose supervisory mechanism and training process are described below.

When the input weights and biases between the input layer and the hidden layer, generated by the SC-III algorithm randomly, satisfies the constraint condition (2), the method has universal approximation property.

(2) $${\langle e_{L-1}{}_{,q},g_{L}\rangle }^{2}\geq b_{g}^{2}\delta_{L,q},q=1,2,\ldots ,m$$

where *e_L_*_−1_ represents the residual error with *L* − 1 hidden nodes; *g_L_* is an activation function, where the Sigmoid function is used, and $0<\left\|g\right\|<b_{g}$ for some *b_g_* ∈ ${\mathbb{R}}^{+}$; *δ_L,q_* represents the *q*th node of the output layer with *L* hidden layer nodes; *m* represents the number of nodes in the output layer.

The training process of the SC-III algorithm mainly includes two phases: hidden layer node parameters configuration and output weight evaluation. In hidden node parameters configuration phase, the connection weight *ω_L_* and bias *b_L_* of the *L*th hidden node are randomly assigned from [−*λ*, *λ*]*^d^* and [−*λ*, *λ*] and the new random basis function *g_L_*(*ω_L_* and *b_L_*) is generated to satisfy (2); the *L*th hidden node output *h_L_* and *ζ_L,q_* are evaluated by (3) and (4), and find the connection weight ω_L_^*^ and b_L_^*^ that maximizes $\zeta_{L}=\sum_{q=1}^{m}\zeta_{L,q}$.

(3) $$h_{L}(X_{S})={\left[g_{L}(\omega_{L}^{T}x_{S1}+b_{L}),g_{L}(\omega_{L}^{T}x_{S2}+b_{L}),\ldots ,g_{L}(\omega_{L}^{T}x_{SN}+b_{L})\right]}^{T}$$

(4) $$\zeta_{L,q}=\left(\frac{{\left(e_{L-1,q}{\left(X_{S}\right)}^{T}\cdot h_{L}(X_{S})\right)}^{2}}{h_{L}{\left(X_{S}\right)}^{T}\cdot h_{L}(X_{S})}-(1-r-\mu_{L})e_{L-1,q}{\left(X_{S}\right)}^{T}e_{L-1,q}(X_{S})\right)$$

where *X_S_* = {*x_S_*_1_, *x_S_*_2_, …, *x_SN_*} is the input matrix of the network, *N* represents the number of training samples, *μ_L_* is a non-negative real number sequence with $\lim_{L\to +\infty }\mu_{L}=0$, 0 < *r*<1, and *μ_L_* ≤ (1 − *r*).

In the weight evaluation phase, the output weights and residual error of SCNs are calculated by (5) and (6).

(5) $$\beta *={\left[\beta_{1}^{*},\beta_{2}^{*},\ldots ,\beta_{L}^{*}\right]}^{T}:=H_{L}^{+}T$$

(6) $$e_{L}=e_{L-1}-\beta_{L}^{*}h_{L}^{*}$$

where $H_{L}^{+}$ is the Moore–Penrose generalized inverse of *H_L_*, *H_L_* = {*h*_1_^*^, *h*_2_^*^, …, h_L_^*^} is the output matrix of the hidden layer, *T* = {*t*_1_, *t*_2_, …, *t_N_*} is the output matrix of the network.

The above two phases are repeated until ${\left\|e_{L}\right\|}_{F}$ (${\left\|•\right\|}_{F}$ is the Frobenius norm.) is less than the preset error and the training ends.

## 4. Incinerator Temperature Prediction Model Based on SCNs

From the above analysis, mutual information, genetic algorithms and SCNs are used to select the feature variables of the MSW incinerator temperature, and the SCN-based incinerator temperature prediction model was established. Thereby, the training efficiency and generalization ability of the prediction model can be guaranteed. In the following section, modeling strategy, hybrid selection method of feature variables and algorithm steps are discussed in detail.

### 4.1. Modeling Strategy

The modeling strategy of predicting the MSW incinerator temperature is that the prediction model is obtained through two-step feature selections. First, MI values between the feature variables of three sub-processes (including grate speed, grate temperature and air flow) and incinerator temperature are calculated, respectively, and feature variables are ranked by MI values size. Next, MI threshold is calculated according to the influence factor of each sub-process, whereby primary feature selection is finished, and feature variables of each sub-process are combined. Then, the secondary feature selection based on the GA-SCN method is conducted, the root means square error (*RMSE*) of incinerator temperature, obtained by training the SCN model, is defined as the fitness function of GAs, and wrapper selection of feature variables is achieved by iterative search from the merged feature variables. In order to avoid the GAs search falling into the local optimum, the search process is repeated *J* times, and the optimal feature subset and the SCN incinerator temperature model with the smallest *RMSE* are obtained through statistical analysis.

### 4.2. Hybrid Selection Method of Feature Variables

The following introduces the implementation method of the primary feature selection based on MI and secondary feature selection based on GA-SCN.

#### 4.2.1. Primary Feature Selection Based on MI

From the analysis of the incineration process in Figure 1, the total number of feature variables that may affect the incinerator temperature from three sub-processes (including grate speed, grate temperature and air flow) are *n*. Take the grate speed sub-process as an example, where there are *n*_1_ initial feature variables. According to the MI formula provided by the literature [27], MI value between the *i*th feature variable $x_{1}^{i}$ and the incinerator temperature *y* is expressed as $I_{1}^{1sti}(x_{1}^{i};y)$, which is calculated as follows:

(7) $$I_{1}^{1sti}(x_{1}^{i};y)=\int \int p(x_{1}^{i}y)\log\frac{p(x_{1}^{i},y)}{p(x_{1}^{i})\cdot p(y)}dx_{1}^{i}dy=H(x_{1}^{i})-H(x_{1}^{i}|y)$$

where $p(x_{1}^{i})$ is the edge probability density with $x_{1}^{i}$, *p*(*y*) is the edge probability density with *y*, $p(x_{1}^{i},y)$ is the joint probability density, $H(x_{1}^{i})$ is the information entropy of $x_{1}^{i}$, and $H(x_{1}^{i}|y)$ is the conditional entropy.

After the MI value of all the feature variables and *y* in the sub-process is calculated, respectively, it is arranged in descending order according to the MI value and the threshold *θ*_1_ is set by (8), and the feature variable with MI value greater than or equal *θ*_1_ is retained, otherwise deleted; thereby the feature subset of the grate speed sub-process $X_{1}^{1st}$ is obtained.

(8) $$\theta_{1}=\frac{\sum_{i=1}^{n_{1}}I_{1}^{1sti}(x_{1}^{i};y)}{n_{1}\cdot \alpha_{1}}$$

where *n*_1_ represents the quantity of initial feature variable in grate speed sub-process; *α*_1_ represents the influence factor of the grate speed sub-process on incinerator temperature, which is set according to the MI value average of each feature variable to incinerator temperature in the three sub-processes. If the MI value average of the sub-process is the smallest, then 0 < *α*_1_ < 1; if it is the largest, let *α*_1_ > 1. If it is centered, let *α*_1_ = 1. As can be seen, the larger the influence factor *α*_1_, the smaller the number of features that are eliminated.

For grate temperature and the air flow sub-processes, MI values between feature variable and *y* are calculated and primary feature selection process is the same as above, and the feature subset $X_{2}^{1st}$ and $X_{3}^{1st}$ can be obtained, respectively. Finally, the selected features are combined by (9) to obtain the feature subset *X*^1*st*^ = {*x*_1_, …, *x_z_*, …, *x_n_*’} after the primary feature selection, and the total number of feature variables is changed from *n* to *n’*.

(9) $$X^{1st}=X_{1}^{1st}\cup X_{2}^{1st}\cup X_{3}^{1st}$$

#### 4.2.2. Secondary Feature Selection Based on GA-SCN

**Figure 2:**
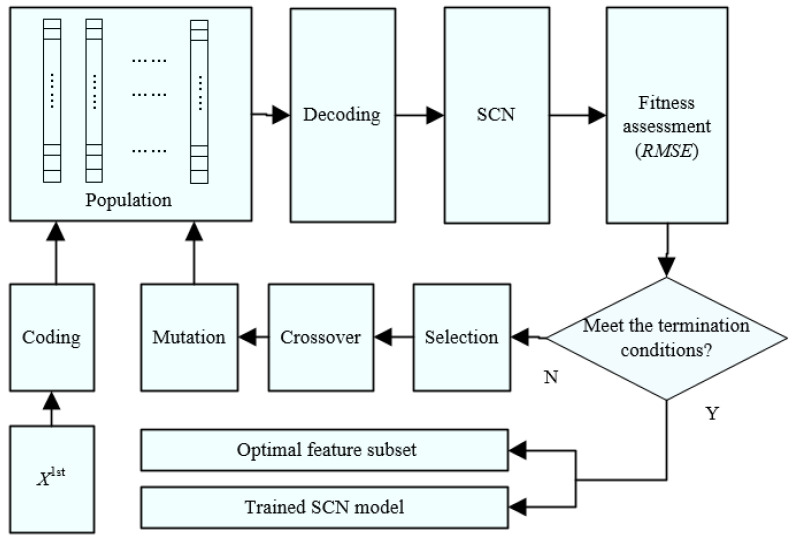
Secondary feature selection procedure based on GA-SCN.

The primary feature selection based on MI only considers the correlation between each feature variable and *y*, however, it ignores the relationship between the variables. Therefore, based on the feature selection of MI, this section combines the GAs [28] proposed by Holland with the SCNs [9] provided by Wang to form a wrapper method for GA-SCN. The procedure is shown in Figure 2. First, feature variables in the feature subset *X*^1*st*^ after the primary selection are coded, and initial population constitutes chromosomes. Then chromosomes in the population are decoded to obtain the feature subset, and *RMSE* is obtained by training the SCN model for assessment fitness, and a new generation of population is formed through the selection, crossover and mutation of chromosomes. The above process is repeated until the SCN model that meets the accuracy requirements is finally obtained.

(1) Coding and decoding

In order to facilitate the implementation of the subsequent evolution steps, the feature subset *X*^1*st*^ needs to be coded, and the coded feature subset is represented in the form of a chromosome vector. The coding rule is as follows:

(10) $$C_{s}^{t}=[c_{1},\ldots ,c_{z},\ldots ,c_{n\prime }]$$

where $C_{s}^{t}$ is the *t*th chromosome in the *s*-th generation population, *n’* is the number of features after the primary feature selection, *c_z_*∈{0,1} is the *z*-th bit of the chromosome (whether the *z*-th feature variable in *X*^1*st*^ is selected for encoding), “0” indicates that the feature variable corresponding to the bit is not selected, and “1” indicates that the feature variable corresponding to the bit is selected.

Based on the above rules, the first-generation population is randomly generated. Among them, *Popsize* is the number of chromosomes, and each bit of the chromosome is randomly generated from 0 or 1.

Decoding ensures that the values are mapped corresponding to each chromosome to feature subsets, and the corresponding relationship is presented as follows:

(11) $$X_{s}^{t}=[c_{1}x_{1},\ldots ,c_{z}x_{z},\ldots ,c_{n^{\prime }}x_{n^{\prime }}]$$

where $X_{s}^{t}$ is the feature subset corresponding to the *t*-th chromosome of the *s*-th generation population, if *c_z_* = 0, let *c_z_x_z_* = ∅.

(2) Fitness assessment

**Figure 3:**
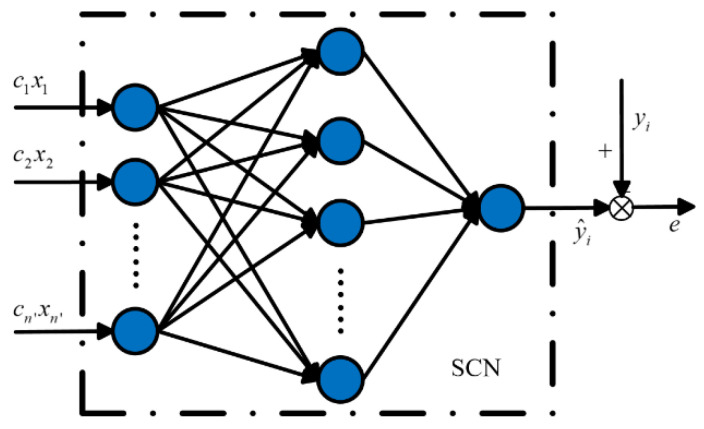
The error of SCN incinerator temperature model.

GAs is a random search strategy based on the theory of “survival of the fittest”. Therefore, the choice of fitness function is a key step of GAs. In this paper, the fitness function is defined by *RMSE* of the incinerator temperature, which is obtained from the SCN model trained by the SC-III algorithm in [9], as shown in Equation (12):

(12) $$fitness_{s}^{t}=\frac{1}{RMSE}$$

where *RMSE* is the root mean square error (*RMSE*) of the SCN model output and the actual value of incinerator temperature, which is shown as follows:

(13) $$RMSE_{s}^{t}=\sqrt{\frac{\sum_{i=1}^{N}{\left(y_{i}-{\hat{y}}_{i}\right)}^{2}}{N}}$$

where *y_i_* is the actual value of the incinerator temperature, ${\hat{y}}_{i}$ is the output of the SCN temperature model in Figure 3.

(3) Selection, crossover and mutation

Selection, crossover and mutation are the main methods of population evolution. The main method of chromosome selection is to select the chromosome with higher fitness as the next generation from the population so as to improve the search efficiency. The selection of chromosomes follows the roulette method so that the more adaptive chromosomes are selected to enter the next generation population with a higher probability. The probability that the *t*-th chromosome in the *s*-th generation population enters the next generation is as follows:

(14) $$p_{s}^{t}=\frac{fitness_{s}^{t}}{\sum_{t=1}^{Popsize}fitness_{s}^{t}}$$

The main role of chromosome crossover and mutation is to ensure the diversity of candidate feature subsets and avoid premature convergence. The one-point crossover method is adopted for crossover, i.e., the intersection point is randomly set in the chromosome. When the chromosome crossover is performed, the anterior and posterior segments of the intersection on two chromosomes are exchanged with the probability *P_c_*. Moreover, mutation occurs when one or more gene positions on a chromosome are selected by the mutant factor randomly and perform a reverse operation with a probability *P_m_*. After the number of iterations *k* reaches the set value, the SCN incinerator temperature model with the smallest *RMSE* and the corresponding feature subset are obtained.

### 4.3. Algorithm Steps

From the above, the specific algorithm steps are described as follows:

Step 1. Parameter initialization, including setting the number of chromosomes *Popsize*, population iteration times *k*, crossover probability *p_c_*, mutant probability *p_m_*, maximum number of hidden nodes *L_max_* of SCN, and GA-SCN program execution times *J*, and then standardize the data;
Step 2. Calculate MI value between each feature variable and incinerator temperature *y* by (7), respectively, and set the influence factors *α*_1_–*α*_3_ of the three sub-processes;
Step 3. Calculate the threshold *θ* of each sub-process by (8). Feature variable is retained if its MI value is greater than or equal to *θ*, otherwise delete it. The selected feature variables in each sub-process are merged into *X*^1st^ by (9);
Step 4. If *J* is equal to 0, go to Step 10, otherwise go to Step 5;
Step 5. Encode the feature variables in *X*^1st^ by (10) and randomly generate the first generation population;
Step 6. Decode each chromosome by (11), train the SCN model according to the SC-III algorithm provided in [9], calculate the fitness of each chromosome by (12), and rank them according to fitness;
Step 7. If error of SCN is less than the expected error, save the optimal feature subset and the corresponding SCN incinerator temperature model, and let *J* = *J* − 1, return to Step 4; otherwise go to Step 8;
Step 8. Calculate the probability that the chromosome is selected to enter the next generation by (14);
Step 9. Perform crossover and mutation of chromosomes based on crossover probability *P_c_* and mutant probability *P_m_* to construct a new generation population and return to Step 5;
Step 10. Compare the feature subsets of the *J* times GA-SCN outputs and use the highest frequency of the same feature subset as the final feature subset, and randomly select the SCN model corresponding to one of the feature subsets as the final incinerator temperature model.

## 5. Experimental Study

### 5.1. Experimental Design

For convenience, the following abbreviations are used: MI is a filter method; GA-SCN is a wrapper method; MI-GA-SCN is the hybrid selection method of feature variables proposed in this paper; BP is the back propagation network algorithm; RBF is radial basis function neural network algorithm.

In order to test the performance of the feature selection method and the incinerator temperature model in this paper, experiments were carried out using historical data of a waste incineration power plant in Beijing in July 2019. The data were standardized by the Z-SCORE standardization method, and the training set and test set were constructed by stratified sampling to ensure the comprehensiveness of the data set. The experimental scheme is as follows:

**Experiment 1:** In order to verify the performance of the hybrid feature selection method MI-GA-SCN proposed in this paper, the running time, number of features and *RSME* of incinerators temperature model were compared for MI, GA-SCN and MI-GA-SCN experimentally.

**Experiment 2:** In order to verify the advantages of SCN applied to modeling for predicting MSW incinerator temperature, the feature subset, obtained by the MI-GA-SCN method, was regarded as the input variable of BP and RBF, and then the incinerator temperature model was obtained by training, respectively. The incinerator temperature model was compared for the SCN, BP and RBF model, and analyzed for *RSME*.

The experimental parameters were as follows: the number of population iterations *k* was set to 10, the number of chromosomes in the population *Popsize* was 20, the mutant probability *P_m_* was 0.05, the crossover probability *P_c_* was 0.4, and the maximum number of hidden nodes *L_max_* of SCN was 500. The GA-SCN program execution times *J* was set to 10.

### 5.2. Analysis of Experimental Results

According to Experiment 1, the feature variables of MSW incinerator temperature were selected by MI, GA-SCN and MI-GA-SCN, respectively, and the running time, the number of selected features and the *RMSE* of the corresponding SCN incinerator temperature model were compared. The comparison results are shown in Table 2. In terms of running time, the filtering method based on MI has the shortest running time, the wrapper method based on GA-SCN has the longest running time, and the hybrid selection method based on MI-GA-SCN is in the middle. Obviously, this proves that the filter method has an advantage in terms of computational efficiency. Moreover, in terms of *RMSE*, the SCN incinerator temperature model based on MI-GA-SCN has the highest accuracy, the SCN model with MI is the least accurate, and the SCN model obtained by GA-SCN is centered.

**Figure 4:**
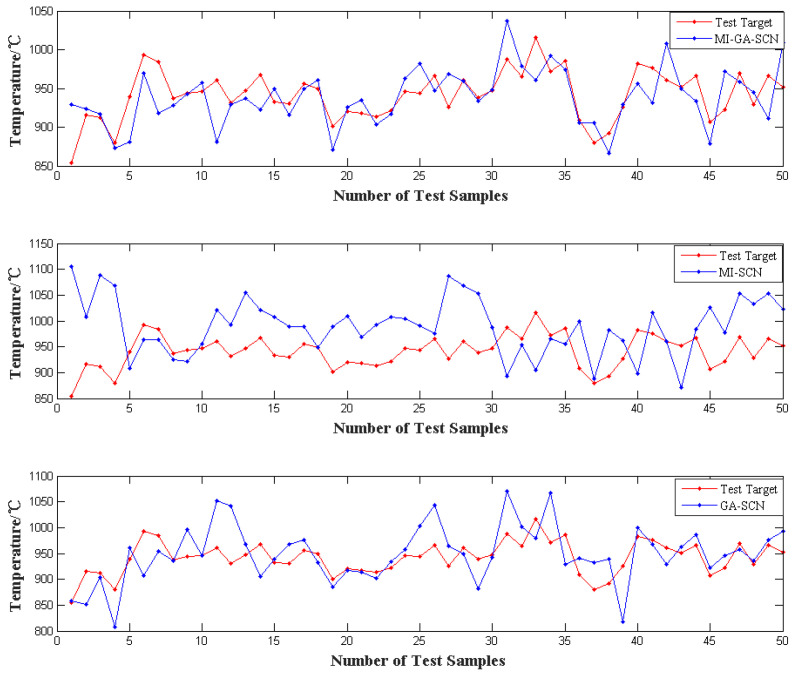
Fitting of incinerator temperature by MI-SCN, GA-SCN and MI-GA-SCN.

In addition, in terms of the number of features, the MI-GA-SCN method selected the least number of features, followed by GA-SCN, and MI selected the largest number of features, which indicates that the combination of filtering and wrapper feature selection methods could be appropriate for making up for their respective shortcomings. The irrelevant variables and redundant variables were eliminated, and the efficiency of feature selection was improved. Simultaneously, the model accuracy significantly improved. Besides, in order to compare the fitting effects of the above three models on the incinerator temperature intuitively, the fitting conditions of the MI-SCN, GA-SCN and MI-GA-SCN incinerator temperature models were compared. The results are shown in Figure 4. It can be seen that the incinerator temperature model obtained by the MI-GA-SCN method can better simulate the change trend of the actual incinerator temperature.

**Figure 5:**
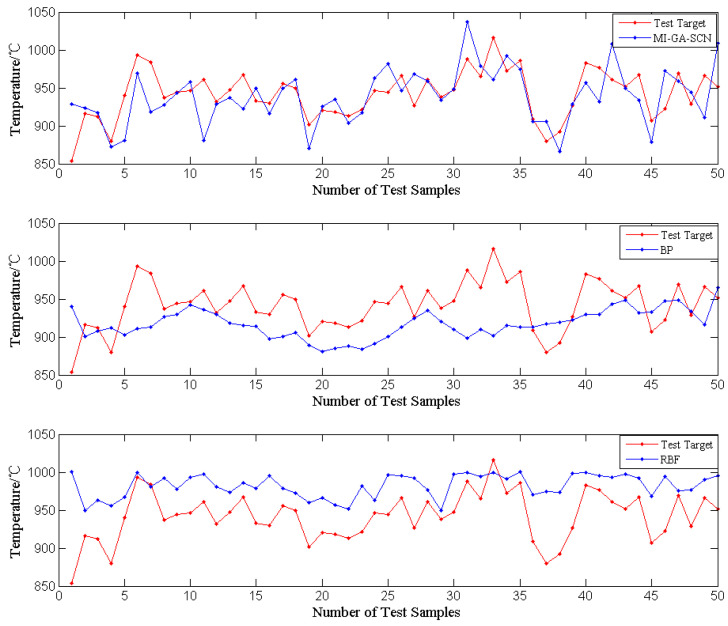
Fitting of incinerator temperature by SCN, BP and RBF.

**Figure 6:**
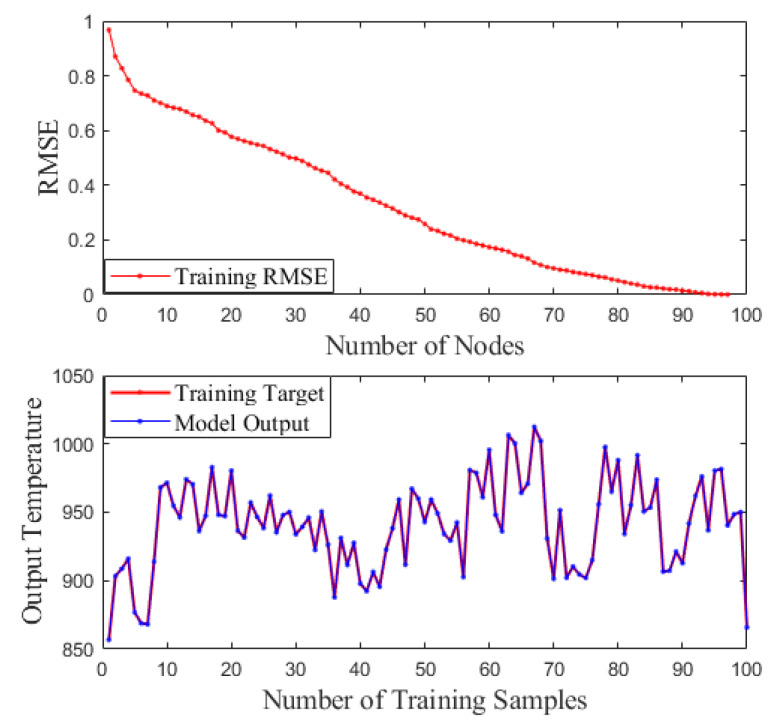
Training situation of SCN model.

The modeling methods are also a key factor affecting the quality of prediction modeling results except for the appropriate feature selection methods. In this paper, the feature subsets, obtained by the MI-GA-SCN method, are used as input variables of the BP and RBF. The BP and RBF incinerator temperature prediction models were obtained by training, respectively, and the SCN incinerator temperature prediction model was obtained by the MI-GA-SCN method. The performance of the above three incinerator temperature models was compared, which is shown in Figure 5. It can be seen that the SCN model is better than the BP model and the RBF model for fitting the incinerator temperature. In addition, Table 3 is the *RMSE* comparison results of the above three models. From the *RMSE* perspective, the prediction accuracy of the SCN model is higher than the BP model and the RBF model. The main reason for the above results is that SCNs have the universal approximation properties for nonlinear functions and the network structure and parameters are configured adaptively, which avoids the influence of artificial experience on neural network learning ability. Moreover, the training situation of the SCN model can also be intuitively seen from Figure 6. This shows that the training *RMSE* of the SCN model reduces to 0 approximately with the hidden layer nodes increasing, which proves that SCNs have advantages in the approximation ability of nonlinear functions again.

In summary, the hybrid selection method of feature variables proposed in this paper could improve the training efficiency and prediction accuracy while reducing the complexity of modeling. In addition, the SCN-based model can predict the trend of MSW incinerator temperature accurately, which indicates that SCNs have certain application advantages in the field of modeling.

## 6. Conclusions

In order to better predict the trend of MSW incinerator temperature, a hybrid selection method for incinerator temperature feature variables and SCN-based prediction incinerator temperature model are proposed in this paper, and the effectiveness of the methods are verified by the historical data of waste incineration process. The main contributions of this method are summarized as follows:

First, in view of the large number of features affecting incinerator temperature in the MSW incineration process, a hybrid feature selection method was proposed. This method combines the MI-based filtering method with GAs-based wrapper method to ensure the efficiency and accuracy of feature selection.

Second, SCN with universal approximation property was used to ensure the accuracy and generalization ability of the incinerator temperature prediction model in the MSW incineration process.

The experimental results indicate that the prediction model proposed in this paper has advantages in training efficiency, prediction accuracy and generalization ability. The reason is that the hybrid selection method effectively eliminates irrelevant variables and redundant variables, and reduces the computational complexity while improving the accuracy of the model. In addition, the advantages of SCNs are the universal approximation property and configuring the network structure and parameters adaptively, which greatly avoids the influence of artificial experience on the learning ability of the neural network prediction model.

Although the experimental results show the rationality of the incinerator temperature model, the model has limitations, such as the inability to process abnormal samples and poor interpretability. Therefore, the directions for future research mainly focus on the modeling strategy combining the mechanism and data to improve the reliability and accuracy of the model, thereby achieving a more reasonable and accurate simulation of the incinerator temperature.

## Figures and Tables

**Table 1:** Details of Feature Variables.

Sub-Process Name	Number of Variables	Details
Grate Speed	18	Feeder velocity (*L*_1_,*L*_2_,*R*_1_,*R*_2_); drying grate velocity (*L*_1_,*L*_2_*,*R*_1_*,*R*_2_); burning grate 1 velocity (*L*_1_,*L*_2_,*R*_1_,*R*_2_); burning grate 2 velocity (*L*_1_,*L*_2_,*R*_1_,*R*_2_); burnout grate velocity (*L*,*R*).
Grate Temperature	24	Drying grate temperature (*L*_1_*,*L*_2_*,*R*_1_,*R*_2_); burning grate 1 inlet temperature (*L*_1_*,*L*_2_,*R*_1_,*R*_2_); burning grate 1 outlet temperature (*L*_1_*,*L*_2_*,*R*_1_*,*R*_2_); burning grate 2 inlet temperature (*L*_1_,*L*_2_,*R*_1_,*R*_2_); burning grate 2 outlet temperature (*L*_1_,*L*_2_,*R*_1_*,*R*_2_*); temperature between drying grate and burning grate (*L*_1_^*^,*L*_2_,*R*_1_,*R*_2_).
Air Flow	23	Drying grate air flow (*L*_1_,*L*_2_*,*R*_1_,*R*_2_); burning grate 1 air flow (*L*_1_*,*L*_2_,*R*_1_*,*R*_2_*); burning grate 2 air flow (*L*_1_*,*L*_2_,*R*_1_,*R*_2_); burnout grate air flow (L*,*R*); fan pressure of primary^*^ and secondary air; air temperature of primary and secondary air heater; secondary air flow; furnace wall cool air temperature (*L*,*R*); furnace grate cool air temperature (L*,*R*).

Remark: In the above table, *L* and *R* represent left and right and the subscripts 1 and 2 represent inside and outside, respectively. In addition, “*” represents the feature variable finally selected by the proposed method.

**Table 2:** Performance comparison of MI, GA-SCN and MI-GA-SCN.

Feature Selection Methods	MI	GA-SCN	MI-GA-SCN
Running time/s	9.7	264.5	225.1
Number of selected features	50	34	19
*RMSE*/°C	85.5129	46.2922	32.2995

**Table 3:** Comparison among *RMSE* (°C) of SCN, BP and RBF.

Number of Experiments	SCN	BP	RBF
1	24.758	43.2929	48.6292
2	37.7678	48.3751	63.5188
3	37.4844	43.0013	59.5881
4	28.8737	41.6697	51.4547
5	34.6255	46.4093	54.4388
6	44.4658	43.1152	64.5210
7	40.4379	46.8904	65.8555
8	40.9450	46.9623	56.0849
Average	36.1678	44.9645	58.0114

## Data Availability

The data presented in this study are available on request from the corresponding author. The data are not publicly available due to privacy.
